# Supplementary material for: The global distribution and spread of the mobilized colistin resistance gene mcr-1
Source: Nat Commun. 2018 Mar 21;9:1179. doi: 10.1038/s41467-018-03205-z (PMC5862964; doi:10.1038/s41467-018-03205-z)
Supplement: Supplementary file 1 — Supplementary Information [file 41467_2018_3205_MOESM1_ESM.pdf]

Supplementary Figures

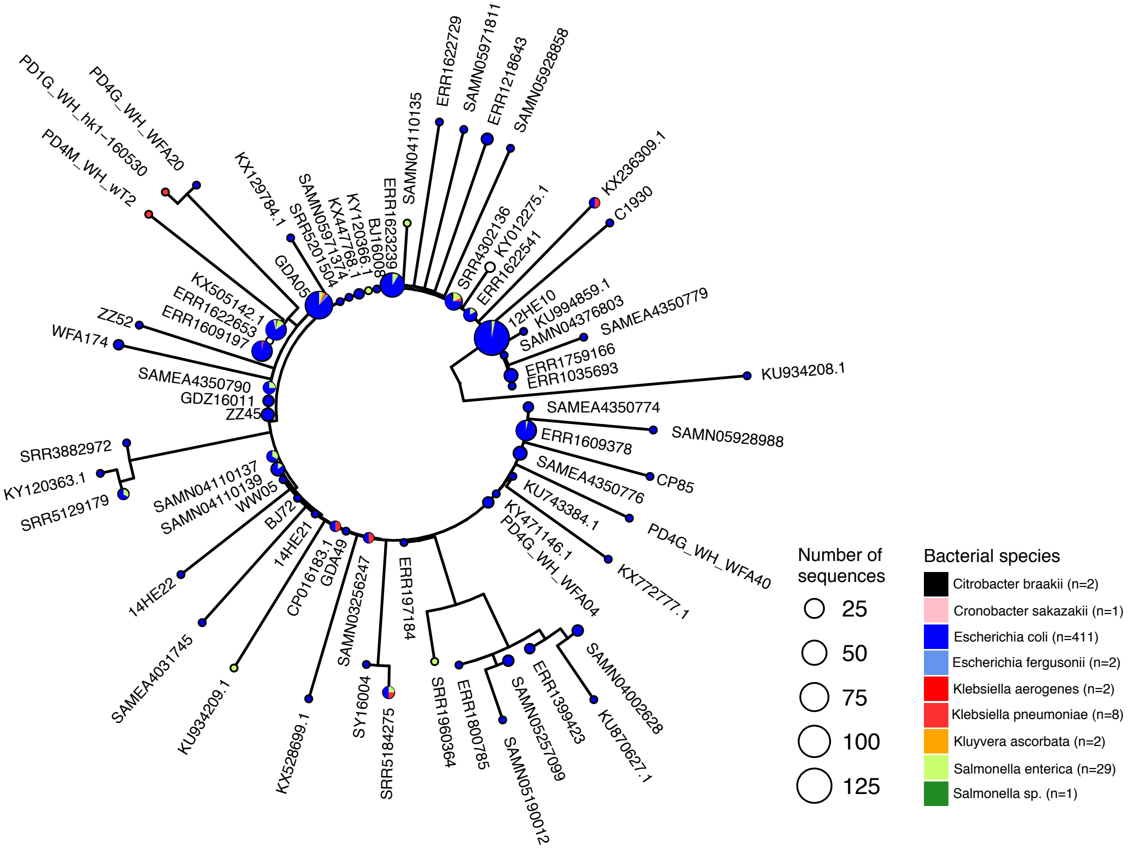

Supplementary Figure 1. The distribution of bacterial host species shown on the transposon phylogeny. Midpoint-rooted maximum parsimony phylogeny based on the 3,522bp alignment of 457 sequences (recombinant regions removed), as in Figures 4, 5, S2. Size of points indicates the number of identical sequences, with a representative sequence for each shown next to each tip. Tips are coloured according to bacterial host species.

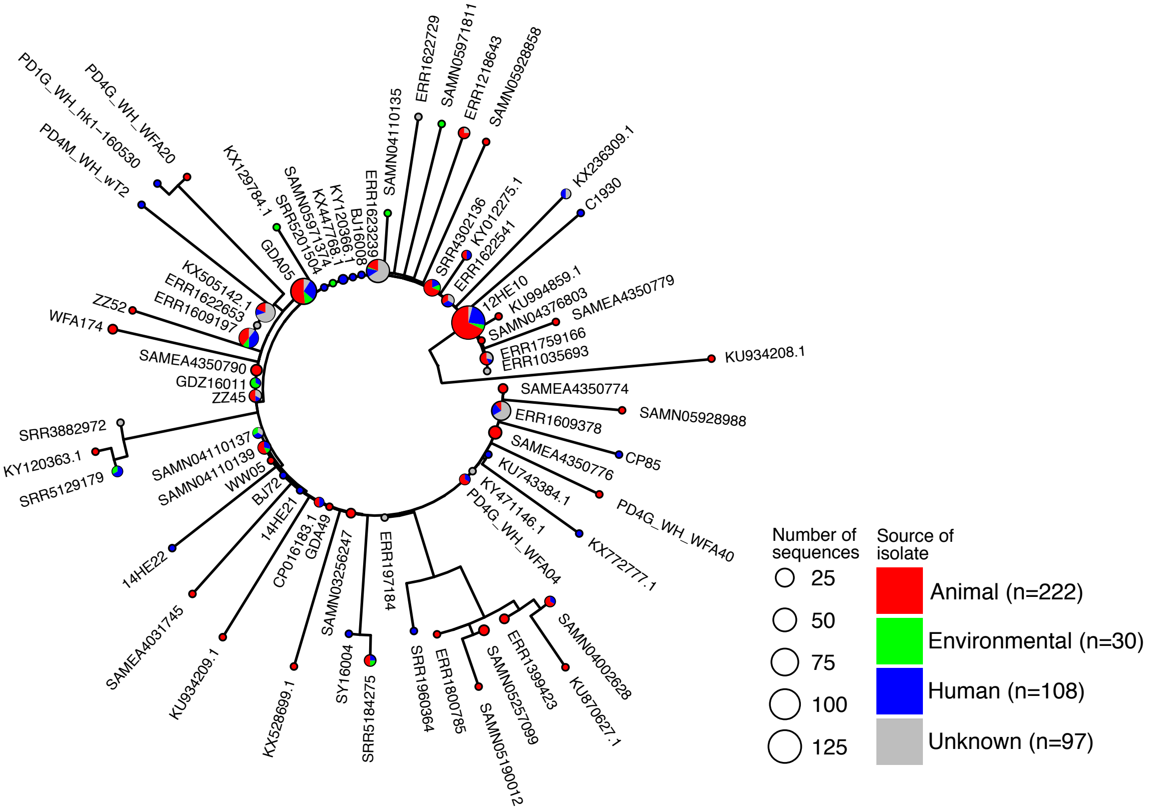

Supplementary Figure 2. The distribution of isolate source shown on the transposon phylogeny. Midpoint-rooted maximum parsimony phylogeny based on the 3,522bp alignment of 457 sequences (recombinant regions removed), as in Figures 4, 5, S1. Size of points indicates the number of identical sequences, with a representative sequence for each shown next to each tip. Tips are coloured according to isolate source.

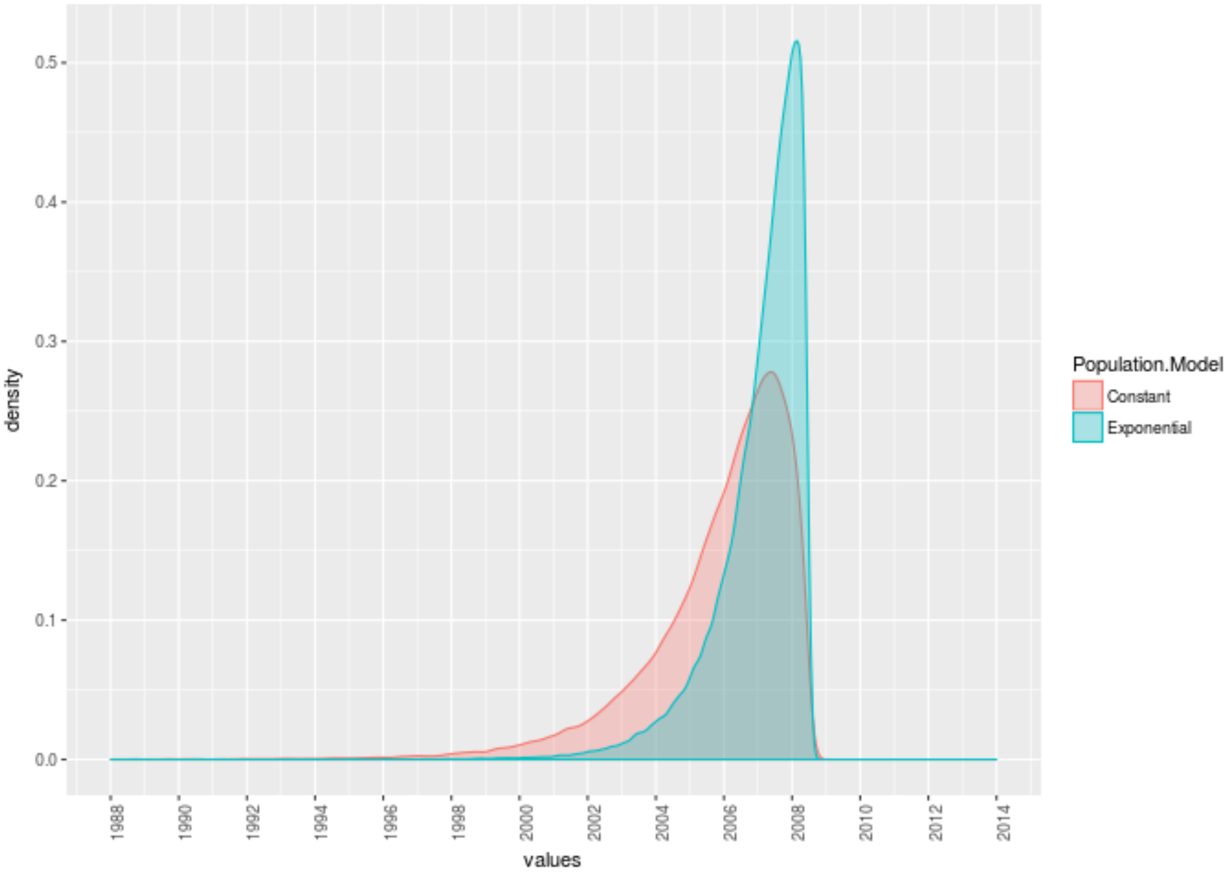

Supplementary Figure 3. Posterior density distributions of root height for the transposon alignment. Distributions under a constant population size model (pink) and a model of exponential population growth (green), both estimated under a strict clock model.

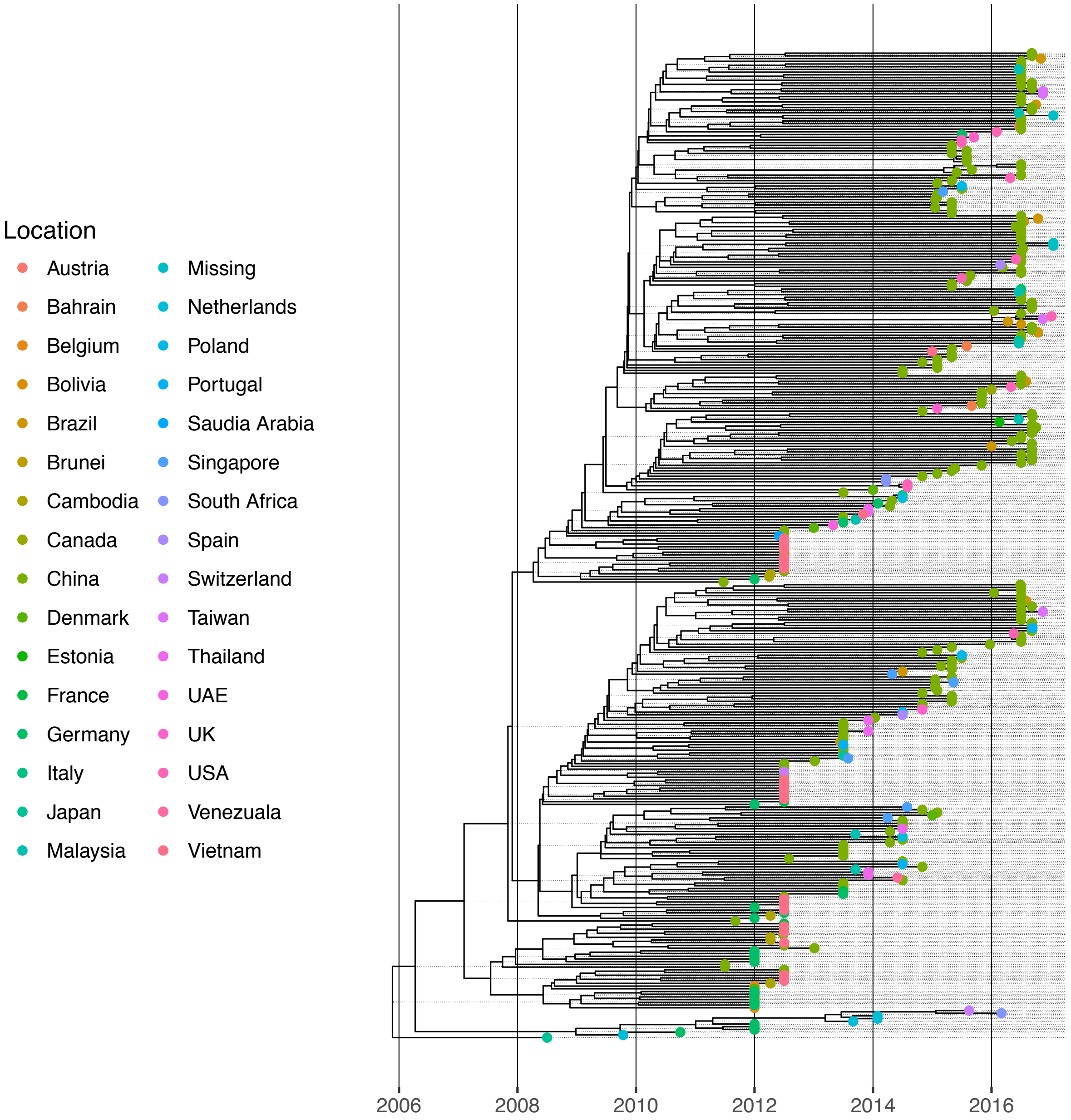

Supplementary Figure 4. Beast inferred maximum clade credibility tree for the *mcr-1* transposon alignment. The timed phylogeny is based on a strict clock model under the coalescent. Tips are coloured according to the country of sampling. The distribution around the root height under different demographic models is provided in Figure S1.

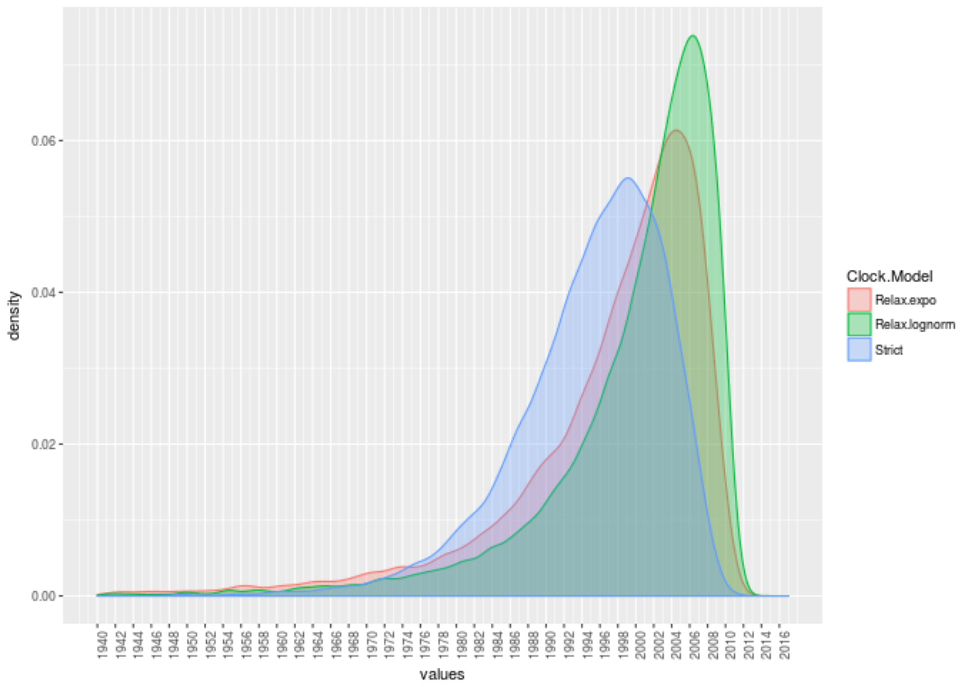

Supplementary Figure 5. Posterior density distributions of root height for the IncI2 plasmid background alignment under different clock models, assuming a constant population size. Different clock models (colours) were used, assuming a constant population size with the Coalescent Bayesian skyline implementation.

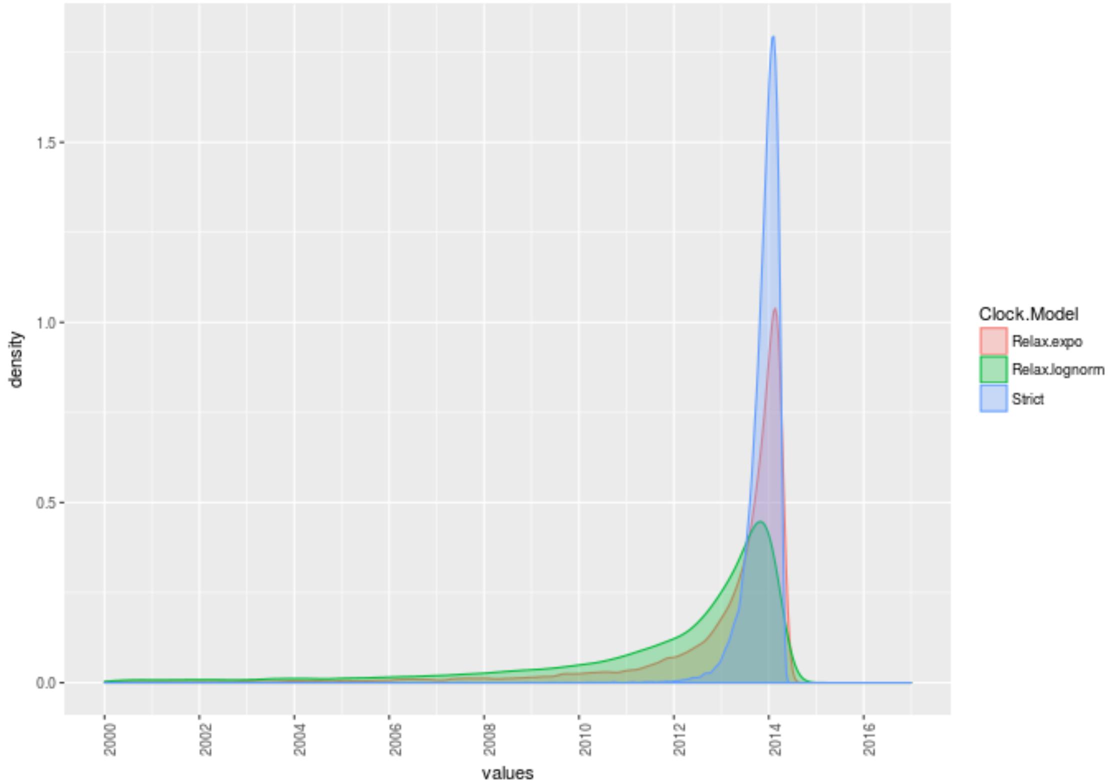

Supplementary Figure 6. Posterior density distributions of root height for the IncX4 plasmid background alignment under different clock models, assuming a constant population size. Different clock models (colours) were used, assuming a constant population size with the Coalescent Bayesian skyline implementation.

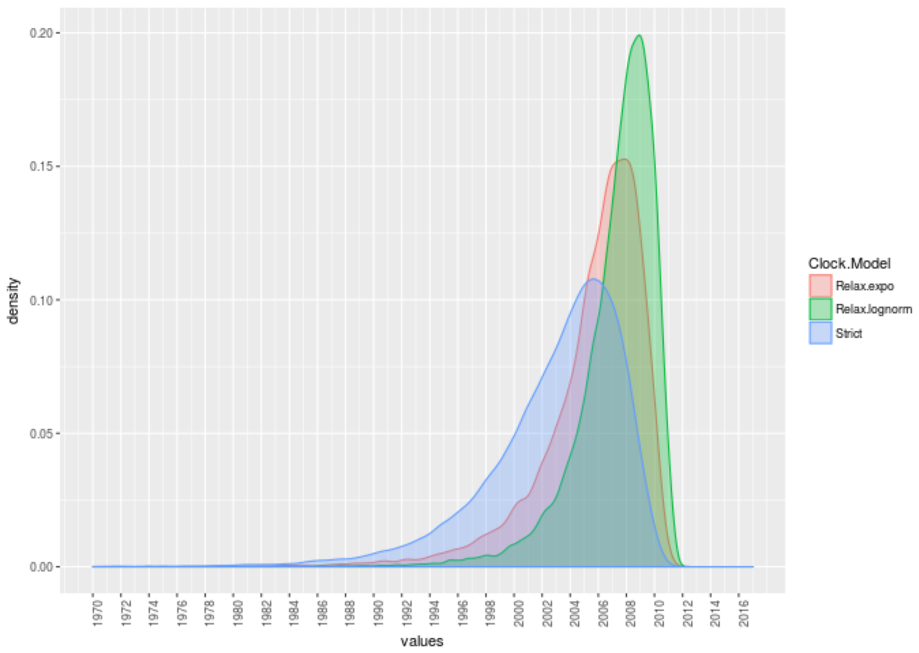

Supplementary Figure 7. Posterior density distribution of root height for the IncI2 plasmid background alignment under different clock models, assuming exponential population growth. Different clock models (colours), assuming a constant population size with the Coalescent Bayesian skyline implementation.

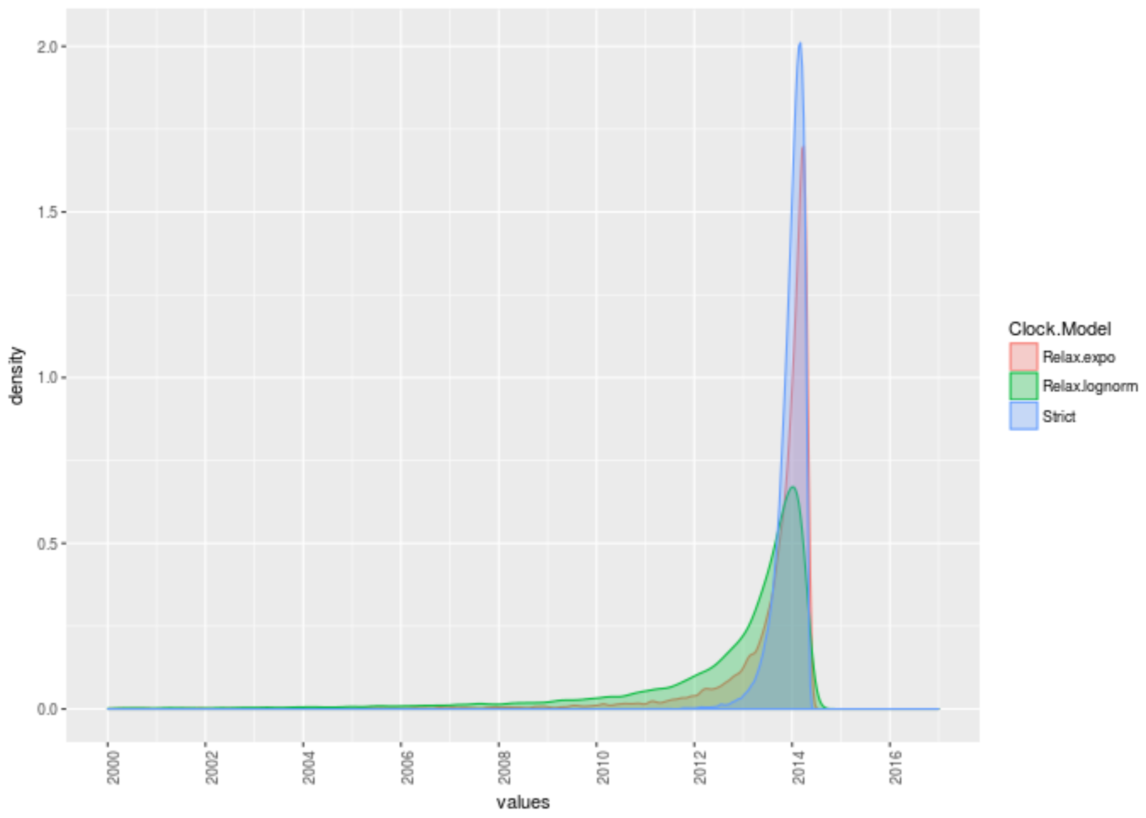

Supplementary Figure 8. Posterior density distribution of root height for the IncX4 plasmid background alignment under different clock models, assuming exponential population growth. Different clock models (colours), assuming a constant population size with the Coalescent Bayesian skyline implementation.

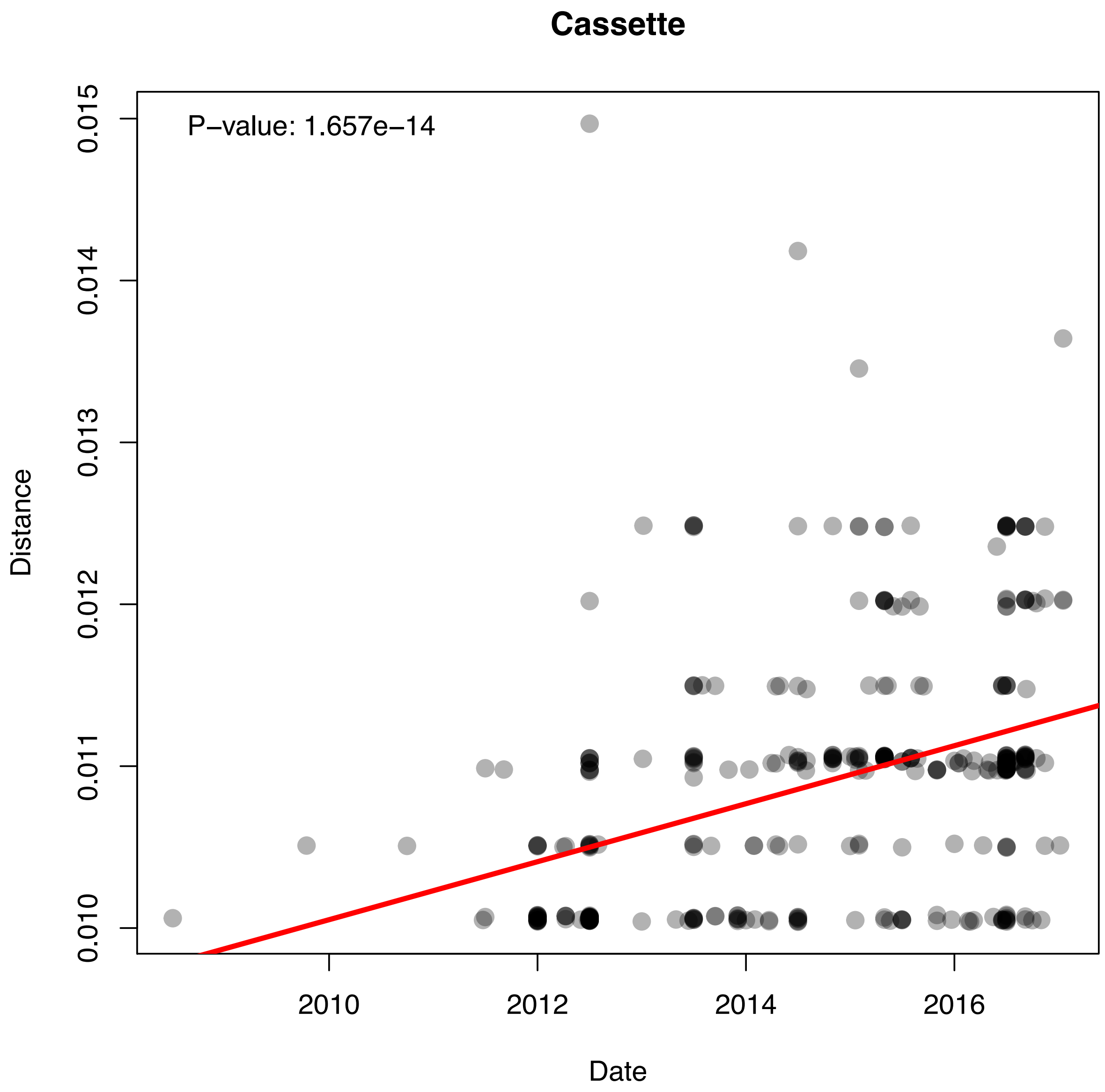

Supplementary Figure 9. Temporal signal in the *mcr-1* transposon alignment (n=457). Root-to-tip distance vs. sampling date.

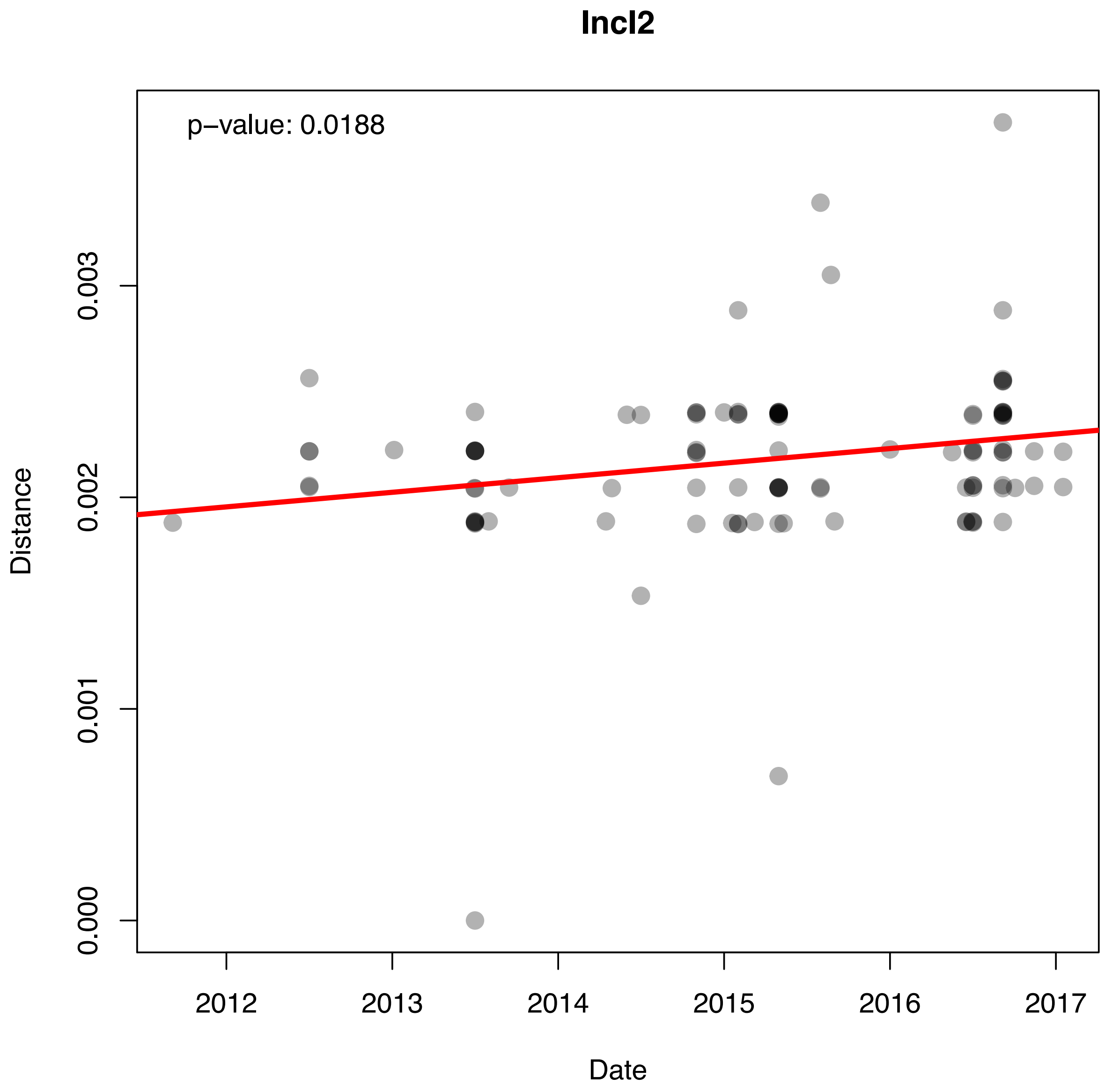

Supplementary Figure 10. Temporal signal in the IncI2 plasmid alignment (n=108). Root-to-tip distance vs. sampling date.

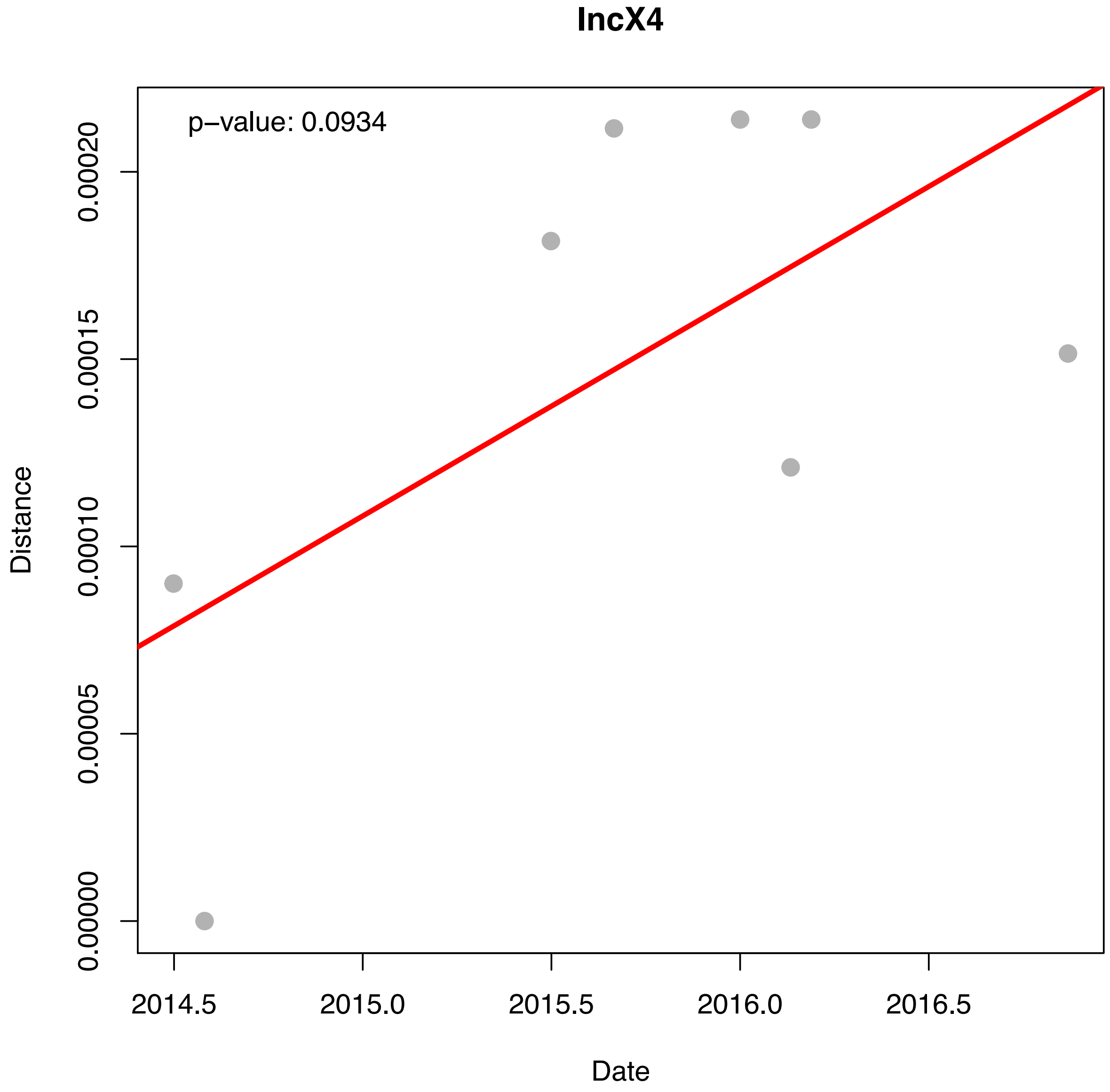

Supplementary Figure 11. Temporal signal in the IncX4 plasmid alignment (n=8). Root-to-tip distance vs. sampling date.

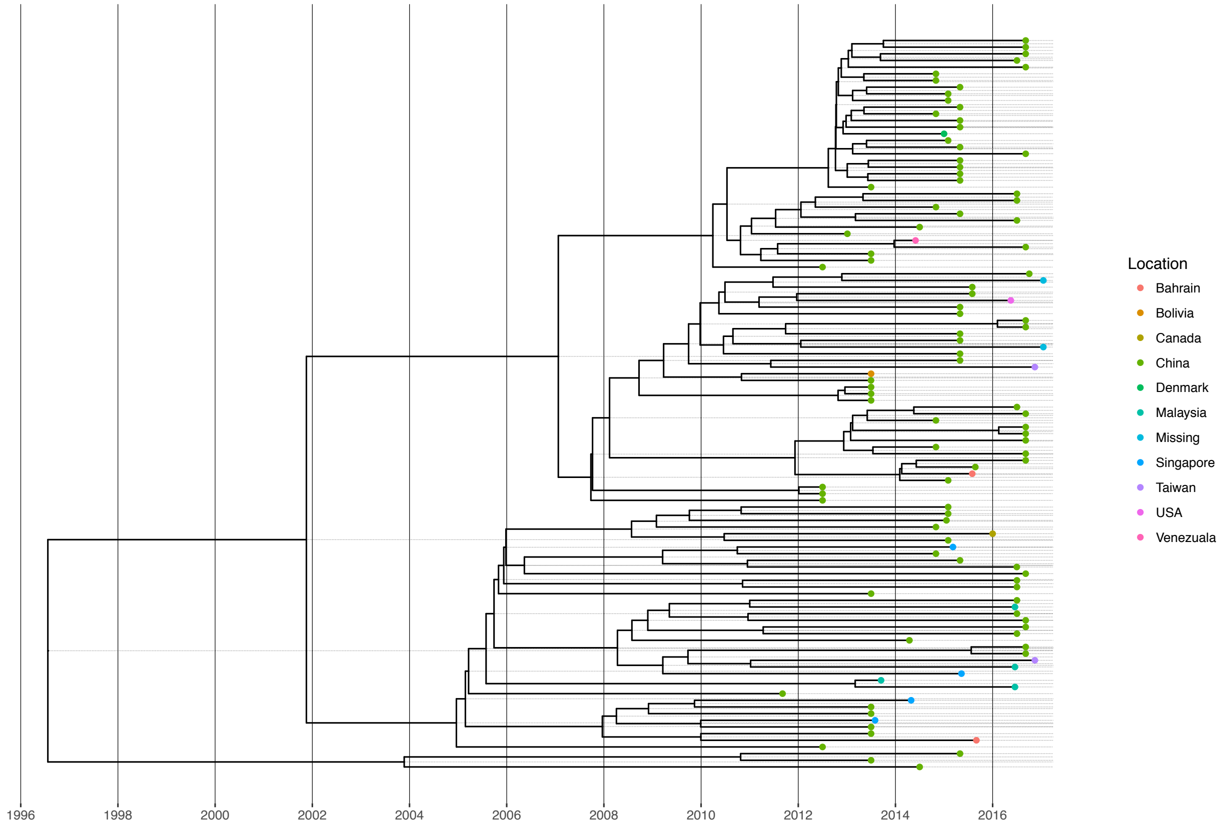

Supplementary Figure 12. Inferred maximum clade credibility tree for IncI2 plasmid backgrounds. The timed phylogeny is based on a relaxed exponential clock under the coalescent model with tips coloured according to the country of sampling. The distributions around the root height under different demographic and clock models are provided in Figures S3 and S4.

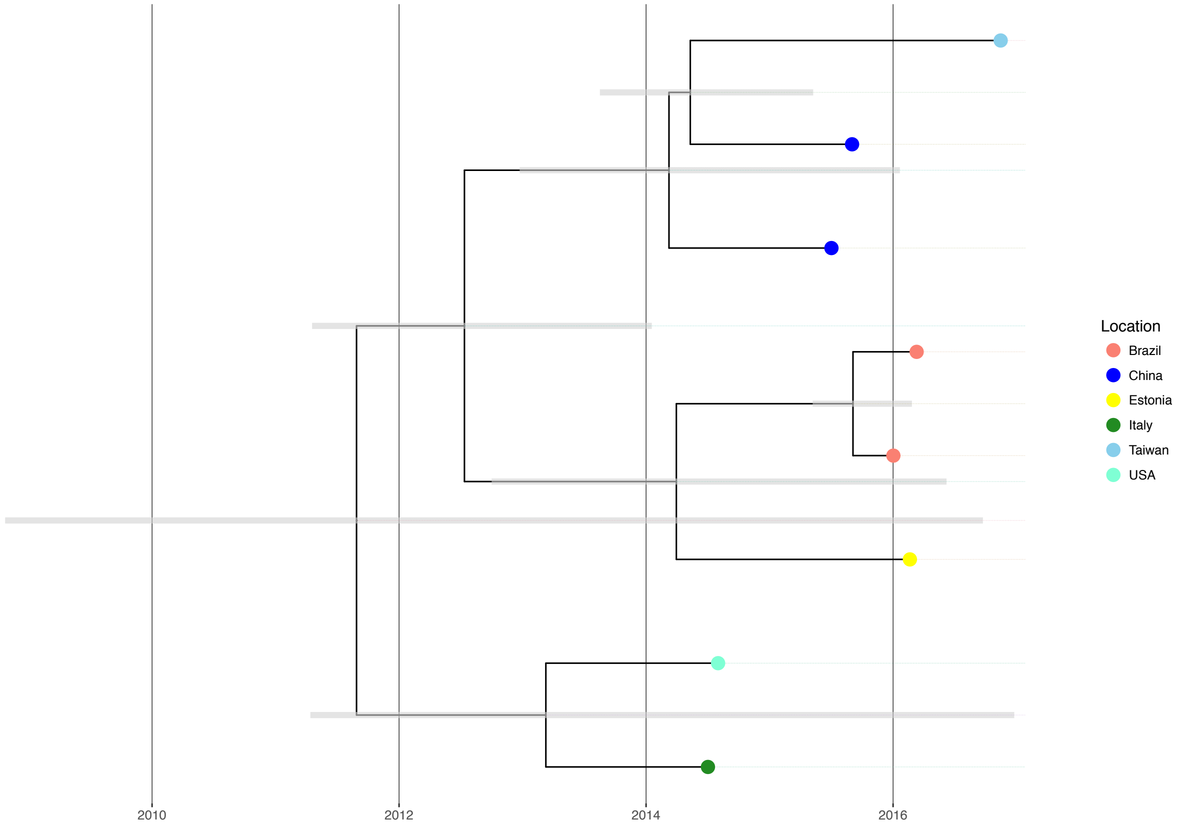

Supplementary Figure 13. Inferred maximum clade credibility tree for IncX4 plasmid backgrounds. The timed phylogeny is based on a relaxed exponential clock under a coalescent model with tips coloured according to the country of sampling. Grey bars give the 95% highest probability density of inferred tree heights at each node. The distributions around the root height under different demographic and clock models are provided in Figures S5 and S6.

Supplementary Tables

Supplementary Table 1. Inferred clock rates expressed as substitutions per base pair per year for the IncI2 and IncX4 plasmid backgrounds together with the *mcr-1* carrying transposon upstream alignment under both constant population size and exponential growth models.

Alignment	Constant Population Size			Exponential Growth		
	Mean	Median	95% HPD	Mean	Median	95% HPD
**IncI2**	5.78E-05	5.66E-05	3.31E-05 - 8.91E-05	6.62E-05	6.46E-05	3.88E-05 - 1.024E-04
**IncX4**	9.48E-05	9.24E-05	4.85E-05 - 1.56E-04	9.78E-05	9.52E-05	5.15E-05 – 1.59E-04
**transposon**	7.55E-05	7.36E-05	4.3E-05 – 1.19E-04	7.50E-05	7.30E-05	4.30E-05 – 1.18E-04

Supplementary Table 2. Model comparison using the AICM approach. The lowest AICMs are highlighted in yellow.

Incl2

Model		AICM	S.E. (+/-)	Model					
				Constant			Exponential		
Population Model	Clock Model			Strict	Relaxed Exponential	Relaxed Lognormal	Strict	Relaxed Exponential	Relaxed Lognormal
Constant	Strict	20711.38	0.2	/	-39.02	-37.07	28.62	-27.81	-36.1
	Relaxed Exponential	20672.37	0.17	39.02	/	1.946	67.63	11.2	2.92
	Relaxed Lognormal	20674.314	0.35	37.07	-1.95	/	65.69	9.26	0.97
Exponential	Strict	20740	0.15	-28.62	-67.63	-65.69	/	-56.43	-64.72
	Relaxed Exponential	20683.57	0.12	27.81	-11.2	-9.26	56.43	/	-8.29
	Relaxed Lognormal	20675.29	0.21	36.1	-2.92	-0.971	64.72	8.29	/

IncX4

Model		AICM	S.E. (+/-)	Model					
				Constant			Exponential		
Population Model	Clock Model			Strict	Relaxed Exponential	Relaxed Lognormal	Strict	Relaxed Exponential	Relaxed Lognormal
Constant	Strict	95663.619	0.031	/	-14.946	-9.974	3.214	-11.7	-6.907
	Relaxed Exponential	95648.673	0.028	14.946	/	4.972	18.16	3.246	8.039
	Relaxed Lognormal	95653.645	0.023	9.974	-4.972	/	13.188	-1.726	3.066
Exponential	Strict	95666.833	0.021	-3.214	-18.16	-13.188	/	-14.914	-10.121
	Relaxed Exponential	95651.919	0.025	11.7	-3.246	1.726	14.914	/	4.792
	Relaxed Lognormal	95656.712	0.017	6.907	-8.039	-3.066	10.121	-4.792	/

Transposon

Model		AICM	S.E. (+/-)	Model					
				Constant			Exponential		
Population Model	Clock Model			Strict	Relaxed Exponential	Relaxed Lognormal	Strict	Relaxed Exponential	Relaxed Lognormal
Constant	Strict	10711.94	0.048	/	/	/	3.46	/	/
	Relaxed Exponential	/	/	/	/	/	/	/	/
	Relaxed Lognormal	/	/	/	/	/	/	/	/
Exponential	Strict	10715.399	0.133	-3.46	/	/	/	/	/
	Relaxed Exponential	/	/	/	/	/	/	/	/
	Relaxed Lognormal	/	/	/	/	/	/	/	/
