# Supplementary material for: The global distribution and spread of the mobilized colistin resistance gene mcr-1
Source: Nat Commun. 2018 Mar 21;9:1179. doi: 10.1038/s41467-018-03205-z (PMC5862964; doi:10.1038/s41467-018-03205-z)
Supplement: Supplementary file 3 — Description of Additional Supplementary File [file 41467_2018_3205_MOESM3_ESM.pdf]

Description of Additional Supplementary File

File Name: Supplementary Data 1
Description: Accession numbers and metadata for the 457 *mcr-1*-positive isolates used in the study
